# Unveiling Social Media Content Related to ADHD Treatment: Machine Learning Study Using X’s Posts over 15 Years

**DOI:** 10.3390/healthcare13192487

**Published:** 2025-09-30

**Authors:** Alba Gómez-Prieto, Alejandra Mercado-Rodriguez, Juan Pablo Chart-Pascual, Cesar I. Fernandez-Lazaro, Francisco J. Lara-Abelenda, María Montero-Torres, Claudia Aymerich, Javier Quintero, Melchor Alvarez-Mon, Ana Gonzalez-Pinto, Cesar A. Soutullo, Miguel Angel Alvarez-Mon

**Affiliations:** 1 Psychiatry Department, Osakidetza Basque Health Service, Araba University Hospital, 01009 Vitoria-Gasteiz, Spain; 2 Bioaraba Research Institute, 01009 Vitoria-Gasteiz, Spain; 3 Department of Neuroscience, University of the Basque Country UPV/EHU, 48940 Leioa, Spain; 4 Centro de Investigación en Red de Salud Mental (CIBERSAM), 28029 Madrid, Spain; 5 Institute of Psychiatry, Psychology and Neuroscience, Child and Adolescent Psychiatry Department, Kings College London, London SE5 8AB, UK; 6 Department of Preventive Medicine and Public Health, School of Medicine, University of Navarra, 31008 Pamplona, Spain; 7 IdiSNA, Navarra Institute for Health Research, 31008 Pamplona, Spain; 8 Department of Signal Theory and Communications and Telematic Systems and Computing, School of Telecommunications Engineering, Rey Juan Carlos University, 28943 Madrid, Spain; 9 Department of Medicine and Medical Specialties, University of Alcala, 28805 Alcalá de Henares, Spain; 10 Biobizkaia Health Research Institute, 48903 Bilbao, Spain; 11 Basurto University Hospital, OSI Bilbao-Basurto, 48013 Bilbao, Spain; 12 Department of Psychiatry and Mental Health, Hospital Universitario Infanta Leonor, 28031 Madrid, Spain; 13 Department of Legal and Psychiatry, Complutense University, 28040 Madrid, Spain; 14 Ramón y Cajal Institute of Sanitary Research (IRYCIS), 28034 Madrid, Spain; 15 Immune System Diseases-Rheumatology and Internal Medicine Service, University Hospital Príncipe de Asturias, 28805 Alcala de Henares, Spain; 16 Centro de Investigación Biomédica en Red Enfermedades Hepaticas y Digestivas, 28029 Madrid, Spain; 17 Louis A. Faillace, M.D., Department of Psychiatry and Behavioral, University of Texas Health Science Center at Houston, Houston, TX 77030, USA

**Keywords:** ADHD, Infodemiology, stimulants, non-stimulants, pharmacology, Twitter, social media

## Abstract

**Background:** Public discourse on social media plays an increasingly influential role in shaping health-related perceptions and behaviours. Individuals share experiences, concerns, and opinions beyond clinical settings around different issues. X (formerly Twitter) provides a unique lens through which to examine how different treatments are perceived, used, and debated across diverse communities over time. **Objective:** The study aims to (a) identify the types of ADHD medications mentioned in posts, depending on language and user type; (b) evaluate the popularity of content related to these medications, considering language and user type; (c) analyse temporal changes in the frequency of mentions between 2006 and 2022; and (d) examine the distribution of tweets across different content categories. By addressing these objectives, this study provides insights into public perceptions of ADHD medications, which may help healthcare professionals better understand online discussions and improve their communication with patients, facilitating more informed treatment decisions. **Methods:** An observational study was conducted analysing 254,952 tweets in Spanish and English about ADHD medications from January 2006 to December 2022. Content analysis combined inductive and deductive approaches to develop a categorisation codebook. BERTWEET and BETO models were used for machine learning classification of English and Spanish tweets, respectively. Descriptive statistical analysis was performed. **Results:** Overall, stimulant medications were posted more frequently and received higher engagement than non-stimulant medications. Methylphenidate, dextroamphetamine, and atomoxetine were the most commonly mentioned medications, especially by patients, who emerged as the most active users among the English tweets. Regarding medical content, tweets in English contained more than twice the number of mentions of inappropriate use compared to those in Spanish. There was a high content of online medication requests and offers in both languages. **Conclusions**: In this study, conducted on X, discussions on ADHD medications highlighted concerns about misuse, adherence, and trivialisation, with clear differences between English and Spanish tweets regarding focus and type of user participation. These findings suggest that monitoring social media can provide early signals about emerging trends, helping clinicians address misconceptions during consultations and informing public health strategies aimed at the safer and more responsible use of ADHD medications.

## 1. Introduction

Attention-deficit/hyperactivity disorder (ADHD) is a childhood-onset neurodevelopmental disorder marked by excessive and impairing inattention, hyperactivity, and impulsivity inappropriate for the child’s age [1]. ADHD is highly genetic, with some environmental causes that interact in a complex way [2]. Globally, it affects an estimated 5.3% of children and adolescents, and around 2.8% of adults [3,4]. ADHD places a substantial strain not only on affected individuals but also on their families and broader society, significantly diminishing quality of life and interfering with everyday activities [5]. People with ADHD are more likely to experience a range of adverse outcomes, such as academic underachievement [6], increased risk of injuries and accidents [7], unplanned teenage pregnancies [8], conflict within the family environment [9], and a higher likelihood of engaging in criminal activity or facing incarceration [10]. In addition, up to 60% of children with ADHD experience comorbid conditions throughout their lives, such as mood disorders, anxiety, substance use disorder and an increased risk of suicide [11]. Due to these conditions, diagnosis is complex, and treatment is delayed. Therefore, it is crucial to clearly understand the diagnostic criteria to make an early diagnosis and start treatment.

ADHD treatment involves psychoeducation and behavioural treatment, academic accommodations and pharmacotherapy with stimulants or non-stimulants [12]. Currently, stimulant medications continue to be the first-line pharmacological treatment for ADHD across all ages due to their safety and efficacy in managing ADHD [13]. Conversely, non-stimulant medications are typically considered a second-line treatment, prescribed when stimulants are ineffective, not tolerated or contraindicated [1,14]. The effectiveness of the ADHD stimulant treatment in treating the primary symptoms of ADHD has been well-determined [15], providing an improvement in quality of life [1,16]. It also significantly increases academic achievement and workplace productivity [17,18]. Some studies reflect a lack of cohesion in prescriptions according to clinical guidelines, demanding greater consensus in diagnostic criteria [19].

Despite being generally well-tolerated and safe, misuse and trivialisation of stimulant medications are common, especially among adolescents and young adults, with studies reporting misuse prevalence ranging from 2.1% to 58.7%, depending on the definition [20]. There is also evidence of very low rates of medication adherence, as low as 23% after 12 months [21]. Beyond low adherence, it is also essential to consider the role of pharmacological tolerance. Evidence suggests that up to 24.7% of patients may experience a gradual reduction in clinical efficacy due to the development of tolerance to stimulant medications over time [22].

Among the multiple causes that can explain this inappropriate use, a widespread and current one nowadays is the use of stimulant medications to enhance academic performance, often referred to as “study drugs”, as indicated in several reports [23]. This potential for abuse is significant and concerning, making it necessary to improve regulation measures for the dispensation of these medications. In cases where there is a suspected risk of medication abuse, it is recommended that the prescription of extended-release formulations be considered, as they may have a lower potential for abuse [14]. As such, on the one hand, we have low adherence to medication that is prescribed by a physician, and on the other hand, frequent misuse, either for recreational or cognitive-enhancing purposes. This is where education and bias can play an important role.

Analysing social media content has become a valuable research tool, providing immediate and unfiltered insights into the experiences and perceptions of different types of users, such as patients or healthcare professionals [24,25,26,27]. Platforms like X (formerly Twitter) host real-time, spontaneous discussions, offering a closer view of attitudes towards different diseases or treatments. Specifically, numerous studies have been conducted on X, exploring mental health topics such as perceptions of pharmacological treatments for severe mental disorders, psychotherapies or electroconvulsive therapy [26,27,28,29]. In addition, several investigations have correlated social media activity with real-world clinical events, such as alcohol consumption [30], opioid misuse [31], or suicide deaths [32], showing significant associations between online postings and these outcomes. Research in ADHD has also compared social media behaviour of patients with and without ADHD, revealing distinct patterns: individuals with ADHD reported greater difficulties in concentration, time management, sleep, and drug abuse, alongside more intense negative emotions and higher posting activity, particularly at night [33]. Moreover, some studies have analysed tweets related to non-medical content and adverse effects of methylphenidate, underscoring the importance of considering both clinical and cultural dimensions of online discourse [34]. However, gaps remain in the literature, particularly regarding the classification of user types, temporal analyses of tweet activity, and the linkage of content categories with different user groups.

This study aimed to (a) identify the types of ADHD medications mentioned in tweets, considering both language and user type; (b) evaluate the popularity of content related to these medications across different groups; (c) analyse temporal changes in the frequency of mentions between 2006 and 2022; and (d) examine the distribution of tweets across distinct content categories. By addressing these objectives, this study seeks to provide healthcare professionals with a better understanding of public perceptions, enabling them to engage with patient concerns and align clinical communication with the realities of the general population.

## 2. Materials and Methods

### 2.1. X Data Collection Strategy

We performed a quantitative study, targeting the collection of tweets that mentioned any pharmacological medications approved by the Food and Drug Administration (FDA) and European Medicines Agency (EMA) for ADHD treatment. We used the Twitter Binder research engine (Twitter Binder, Pamplona, Spain) to source these tweets, which allows access to 100% of publicly available tweets. To ensure comprehensive data collection, we first developed a list of keywords that would capture all relevant tweets. This list included the generic and authorised brand names of the medications used to treat ADHD in English and Spanish (keywords can be found in Table S1 of the Supplementary Material). By incorporating these specific keywords, we aimed to cover all possible variations in how these medications might be mentioned on X. We then systematically gathered all tweets containing any of these selected keywords, ensuring that our dataset was as complete and relevant as possible. The inclusion criteria for the tweets were: Tweets publicly available that contained the specific keywords pertinent to the study, published between 1 January 2006 and 31 December 2022, in English or Spanish.

### 2.2. Content Analysis Process

#### 2.2.1. Exploration of Data and Identification of Categories

We utilised deductive and inductive reasoning to develop a categorisation codebook for classifying tweet content into distinct codes based on key thematic categories. We integrated categories identified in our previous research on X for the deductive component [25,35,36]. We used an inductive approach by initially analysing a sample of 500 tweets from a smaller subset chosen for manual classification. This allowed us to identify potential new themes and refine the categorisation codebook. Three researchers (AG, AMR, and JCP) independently coded this subset of tweets to ensure accuracy. They then discussed any discrepancies, reaching a consensus with the mediation of a fourth researcher (MAAM). After finalising the categorisation codebook, two study members (AG and AMR) each coded 3000 tweets in English and Spanish from the databases.

As explained in Table 1, the manual analysis followed a detailed codebook method involving several steps and specific criteria for evaluating each tweet. First, tweets were divided into two initial categories: classifiable and unclassifiable. Classifiable tweets included comprehensible Spanish or English tweets containing meaningful content that addressed neuropsychiatric topics. In contrast, unclassifiable tweets were those written in Spanish or English but vague, lacking sufficient detail, or addressing medical topics unrelated to neuropsychiatry. Once identified as classifiable, tweets were further categorised into two main groups: Type of user (patient, healthcare professional, healthcare institution/academic entity, or undetermined) and content (medical content or other types of content). Within the medical content group, tweets were subdivided by topic: drug efficacy (mentioned or not), drug adherence (mentioned or not), inappropriate use (mentioned or not), side effects (mentioned or not), and psychiatric diagnosis (whether the term ADHD was mentioned or not). The other types of content group included tweets addressing economic and legal activities, advocacy, trivialisation of treatment, requests and offers, or, if none of these applied, were placed in an “undetermined” category.

#### 2.2.2. Machine Learning Classifier

Machine learning plays a pivotal role in analysing large datasets that are too extensive to evaluate manually. As a subset of artificial intelligence, machine learning includes three primary types: supervised, unsupervised, and semi-supervised learning [37]. This research employs semi-supervised learning, which integrates aspects of supervised and unsupervised methods by utilising labelled and unlabelled data. This approach extends traditional manual analysis, aiming to create a model replicating expert evaluations for classifying millions of tweets.

First, the tweets underwent a pre-processing step, which involved normalising them by removing special characters, splitting negative contractions, removing repetitions and transforming emojis into words. Then, the two manually classified databases (Spanish and English), composed of 3000 tweets each, were randomly divided into an 80% training subset (2400 tweets) and a 20% testing subset (600 tweets). The training subset trained one Machine Learning model for each classification category. On the other hand, the testing subset was used to validate the performance of the models. Despite the availability of various pre-trained models for text classification in the literature, we used a transformer-based model known as BERTweet [38] for the English dataset and a model called BETO for the Spanish dataset [39]. BERTWEET is a model based on BERT trained with 80 GB of text containing over 860 million English tweets. The choice of these models is based on their widespread use in the literature [40,41] and their specific training using English tweets similar to the ones we will evaluate. For the Spanish dataset, we used BETO, which is a BERT model trained on a Spanish corpus and has also been extensively used in the literature too [42,43].

The BETO and BERTweet models required fine-tuning for each category to ensure they accurately replicated expert analyses. This process involved adjusting the parameters of a pre-trained model using specific data from the new task [44]. The goal was to leverage the general knowledge acquired by the model during its pre-training on large, unlabelled datasets and adapt it to more specific tasks. In this context, the English version of the manually labelled dataset was employed to fine-tune the BERTweet model. In contrast, the Spanish version of the manually classified tweets was used to fine-tune the BETO model. One challenge during fine-tuning was the imbalance of options within each category. To address this, we used the Easy Data Augmentation (EDA) pipeline to create new tweets, ensuring an equal number of each option within the same category [45]. EDA creates new tweets by replacing words with their synonyms, removing some random words, and switching the positions of words.

Finally, we used the test datasets to check the performance of the fine-tuned models. We used the F1 score to analyse the performance of each model across all the categories. The model performed well in the test set, achieving a mean F1 Score higher than 0.72 in all categories. The categories with a mean F1 score lower than 0.75 were *type of user* (0.72) and *other type of content* (0.72). Conversely, the categories with a mean F1 score higher than 0.85 were *inappropriate use* (0.85) and *drug adherence* (0.89). After verifying the models’ strong performance, they were deployed to classify the remaining tweets.

### 2.3. Statistical Analysis

The statistical methods used in the analysis were descriptive. Data on tweets were summarised using frequencies and proportions stratified by drug for the Spanish and English datasets and for both (Spanish + English combined) datasets. For each dataset, we graphically represented the frequencies of tweets by user type stratified by drug-using bubble plots, the proportion of tweets according to the content topic of the study using a bar chart, and the proportions of tweets by type of content stratified by drug using a heat map. Time trends were additionally represented to describe the number of tweets posted by user type during the study period. All analyses were performed with STATA version 15 (StataCorp LP, College Station, TX, USA).

### 2.4. Ethical Considerations

The present study was reviewed and approved by the Research Ethics Committee of the University of Alcalá (OE 14_2020), and it strictly adheres to the ethical principles of research outlined in the Declaration of Helsinki [46]. As the study utilised publicly available tweets and did not directly involve human subjects or interventions, there was no direct risk to the privacy or safety of individuals. Nonetheless, we took great care to protect the confidentiality of all users by ensuring that no personal identifying information was collected or disclosed in the analysis. Additionally, we were vigilant in avoiding the inclusion of any tweets that may have inadvertently revealed the users’ identities.

## 3. Results

### 3.1. Total Number of Tweets per Drug, Engagement and Temporal Evolution

**Figure 1:**
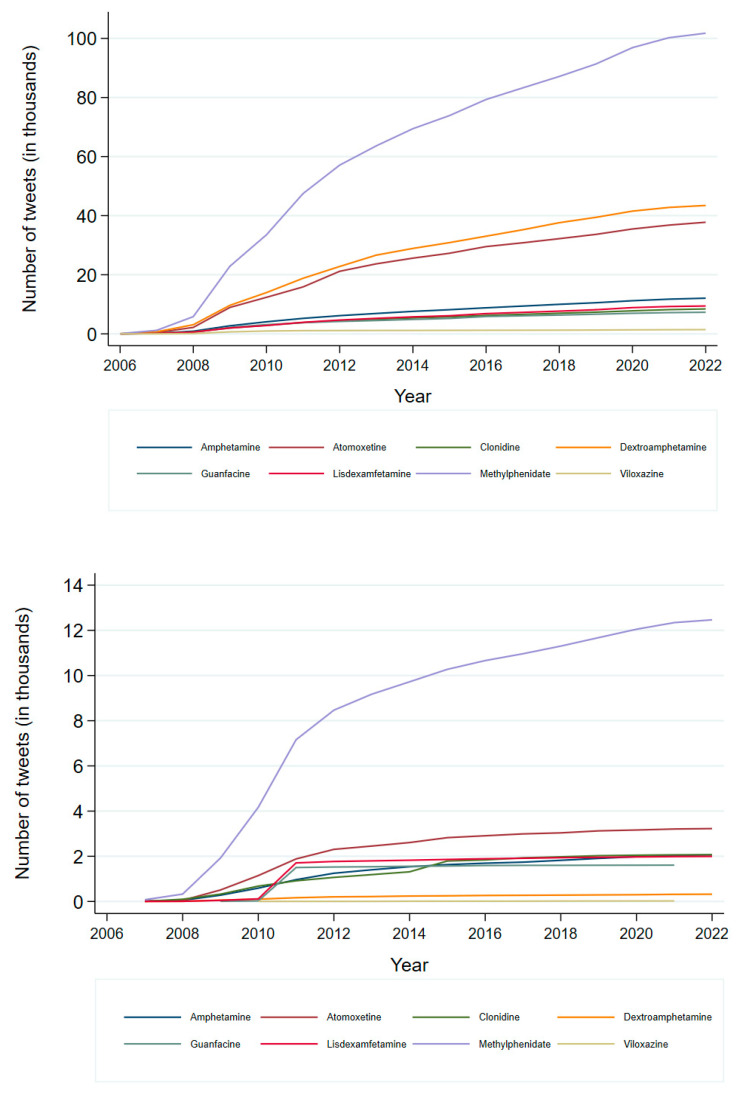
Annual frequency of English (**Top** panel) and Spanish (**Bottom** panel) tweets mentioning stimulants and non-stimulant drugs used for ADHD. Each colour represents a specific drug as indicated by the legend below.

A total of 245,467 tweets were included in the study, with 90.3% (221,695/245,467) in English and 9.7% (23,772/245,467) in Spanish. Overall, stimulants were mentioned more frequently in posts than non-stimulants. Specifically, 75.2% of English and 70.9% of Spanish tweets focused on stimulants (Table 2). Regarding the English-posted tweets, the most discussed drug was methylphenidate (45.9%), followed by dextroamphetamine (19.6%) and atomoxetine (17%) (Table 2). These three medications also showed the greatest increase in tweets over the years, as seen in Figure 1. Conversely, Methylphenidate was the drug with the highest number of posted tweets in the Spanish dataset (52.4%), followed by atomoxetine (13.6%) and clonidine (8.7%), as shown in Table 2.

Overall, stimulants had higher engagement than non-stimulants, with higher RT/Tweet and Like/Tweet ratios. (The tweet engagement is measured through Like/Tweet and RT/Tweet ratios, representing the average number of likes and retweets obtained per tweet. These ratios evaluate content popularity and virality based on the interactions received). Dextroamphetamine had the highest Like/Tweet ratio (376.97) in English tweets, while the Like/Tweet ratios of clonidine (1.91) and atomoxetine (1.12) topped the ratios in Spanish tweets.

### 3.2. User Type Analysis

**Figure 2:**
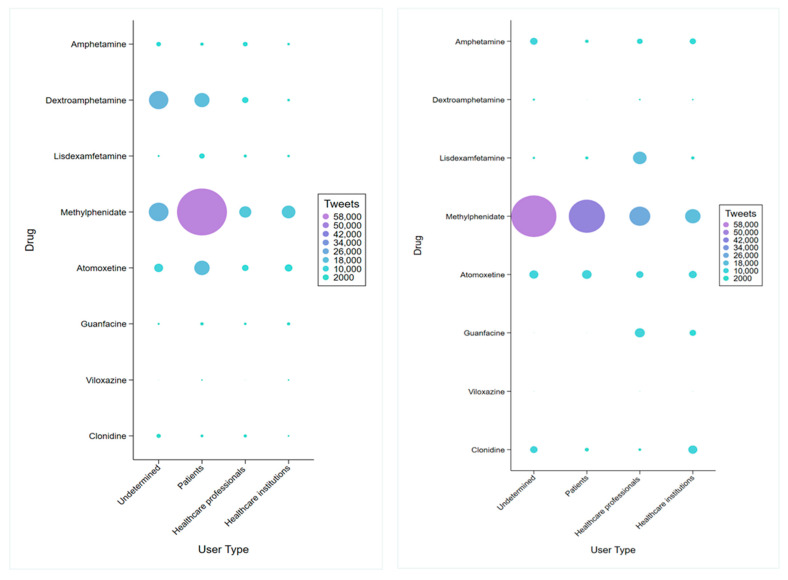
Distribution of tweets mentioning stimulant and non-stimulant drugs used in attention deficit hyperactivity disorder (ADHD), classified by user type. Each circle represents the total number of tweets posted for a given drug-user type combination, with circle size proportional to tweet volume (see scale in legend). The x-axis indicates the type of user (undetermined, patients, healthcare professionals, or healthcare institutions), and the y-axis indicates the specific medication. **Left** panel: Tweets posted in English. **Right** panel: Tweets posted in Spanish.

In English tweets, patients were the most common users (44.4%, n = 98,517), while in Spanish tweets, the type of user was more evenly distributed, including patients (23.75%, n = 5647), healthcare professionals (25.2%, n = 5990), and institutions (19.75%, n = 4696). Methylphenidate was the most posted-about drug among patients in both English (45.9%, n = 101,759) and Spanish (52.44%, n = 12,465) tweets (Figure 2). This pattern was also seen among healthcare professionals, with methylphenidate being the most discussed drug in both languages (11.96%, n = 12,171 in English; 17.42%, n = 2171 in Spanish). In English tweets, healthcare professionals also frequently mentioned dextroamphetamine (13.31%, n = 5786) and atomoxetine (15.94%, n = 6023), while in Spanish tweets, discussions by healthcare professionals were spread across multiple medications, including lisdexamfetamine (68.97%, n = 1376) and guanfacine (60.32%, n = 970). Among English tweets from healthcare institutions, the focus was primarily on methylphenidate and atomoxetine, whereas Spanish tweets from healthcare institutions showed a more even distribution among different medications, as shown in Figure 2.

**Figure 3:**
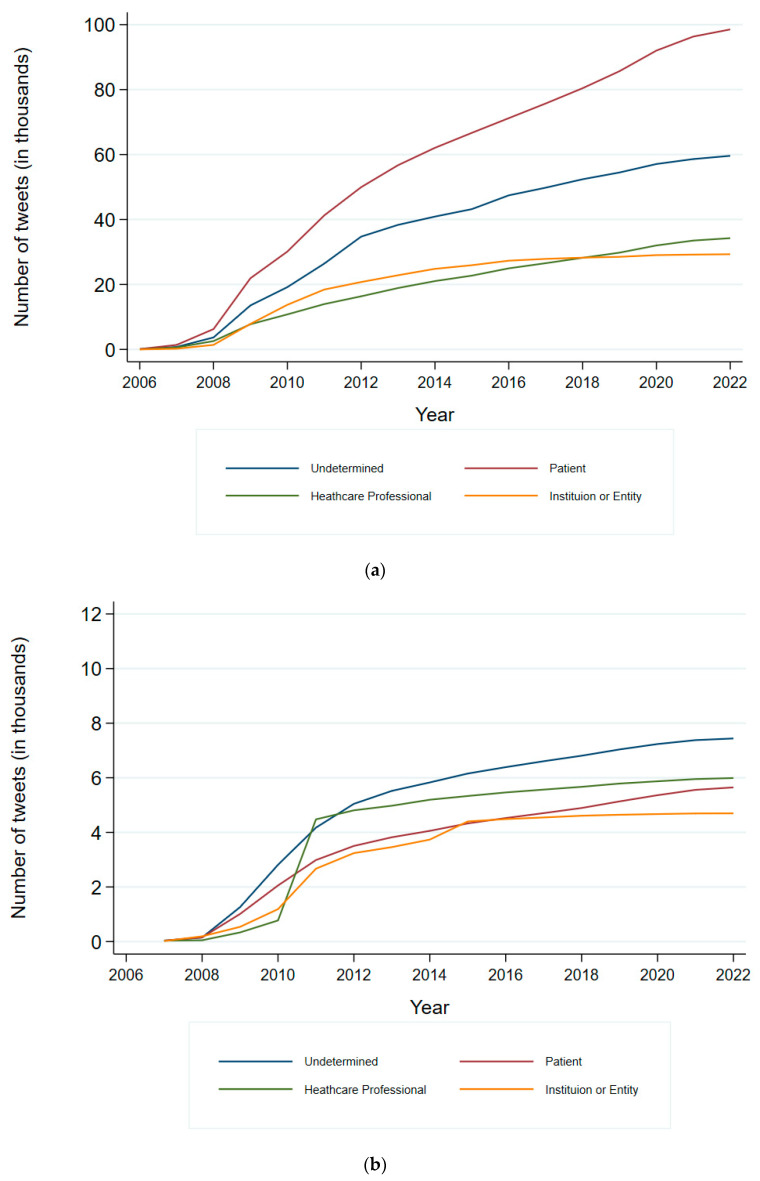
Annual frequency of tweets mentioning stimulant and non-stimulant drugs used for ADHD, stratified by user type. Top panel (**a**) shows tweets published in English, and bottom panel (**b**) shows tweets published in Spanish. The x-axis represents the year of publication (2006–2022), and the y-axis represents the number of tweets expressed in thousands. Each coloured line corresponds to a different user type: Undetermined, Patient, Healthcare Professional, or Institution/Entity, as indicated in the legend.

Related to the activity on X of the different user subgroups, the data showed a steady increase in tweet volume across all user types over the years. In English tweets (Figure 3a), *patients* exhibited the highest growth, with tweet volumes rising rapidly, especially after 2010. Tweets from *healthcare professionals* grew steadily, while those from *institutions or entities increased* more slowly. In Spanish tweets (Figure 3b), *patients and healthcare professionals* exhibit similar growth patterns, with tweet volumes increasing rapidly until 2012 and then continuing to grow at a more gradual pace. Tweets from *institutions or entities* in Spanish also increase steadily but remain the lowest in volume compared to other user types.

### 3.3. Medical Content Analysis

**Figure 4:**
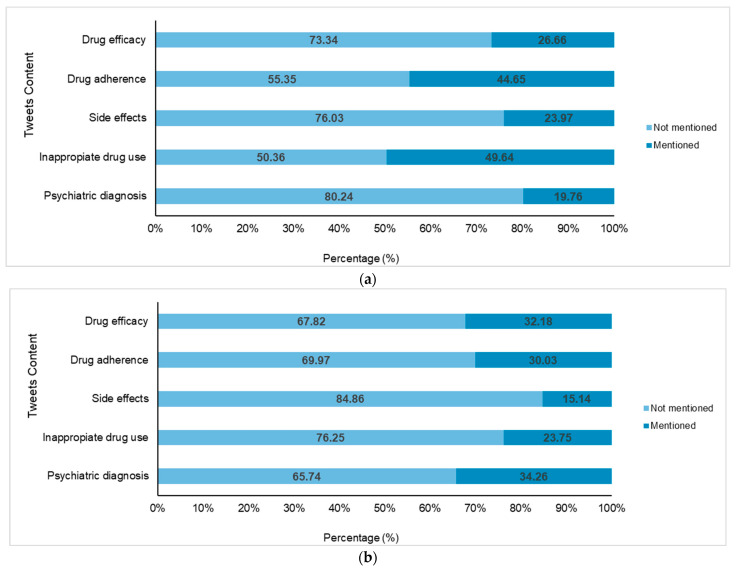
Proportion of tweets posted of stimulant and non-stimulant drugs used in attention deficit hyperactivity disorder (ADHD) according to content topics of the study. The top panel: (**a**) English tweets; the bottom panel: (**b**) Spanish tweets.

Based on the content of the tweets, English tweets (Figure 4a) had a higher percentage of discussions related to inappropriate medication use (49.64%) compared to Spanish tweets (23.75%), as shown in Figure 4b. Similarly, treatment adherence was a prominent topic of discussion, with 44.65% of English tweets covering this compared to 30.03% of Spanish tweets. Both languages displayed similar proportions of mentions regarding drug efficacy, with 26.66% for English and 32.18% for Spanish. Side effects were noted in 23.97% of English tweets but were less frequently mentioned in Spanish in 15.14%. Most notably, a significant contrast was observed in the mentions of psychiatric diagnoses, which were present in only 19.76% of English tweets compared to 34.26% of Spanish tweets.

### 3.4. Other Types of Content Analysis

**Figure 5:**
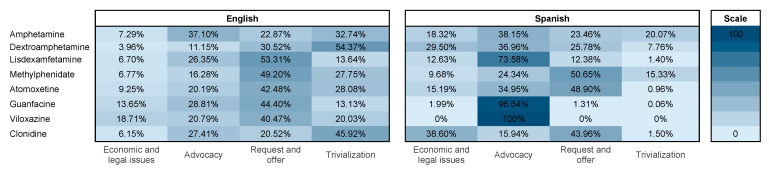
Heatmap explaining percentage of tweets posted in English and Spanish of stimulant and non-stimulant drugs used in attention deficit hyperactivity disorder (ADHD) according to topics of the study.

Based on the data in Figure 5, English tweets showed a significant prevalence of requests and offers related to various medications, with methylphenidate, lisdexamfetamine and atomoxetine being the most discussed medications, representing 53.31%, 49.20%, and 42.48% of the related tweets, respectively. A noteworthy number of tweets also appeared to trivialise the use of these medications, including dextroamphetamine (54.37%), clonidine (45.92%) and amphetamine (32.74%). A prominent presence of advocacy content was also observed, containing notably material related to amphetamine (37.10%) and clonidine (27.41%). Regarding tweets posted in Spanish, they exhibited a substantial amount of advocacy content, particularly regarding the studied medications, notably viloxazine at the forefront (100%), followed by guanfacine (96.64%). Similarly, a substantial percentage of Spanish tweets focused on requests and offers, particularly regarding methylphenidate (50.65%) and atomoxetine (48.90%). Furthermore, there was a notable emphasis on content that trivialised medications, with amphetamine being among the top medications receiving such attention (20.07%), followed by methylphenidate (15.33%). Figure S1 in the Supplementary Material presents a visual summary of the methodology and key results obtained in this study.

## 4. Discussion

In this study, we analysed tweets posted on X over 15 years (2006–2022) that mentioned the approved stimulant and non-stimulant medications for ADHD in both English and Spanish. Our findings indicate a predominance of tweets in English and greater activity surrounding stimulant medications compared to non-stimulants. In English language tweets, Methylphenidate was the most frequently mentioned drug, particularly by patients. This contrasts with the more heterogeneous participation seen in Spanish-language tweets, especially among health professionals. Additionally, there was a higher prevalence of tweets related to the inappropriate use of these medications in English. In both languages, a substantial volume of requests and offers for these medications was observed.

In our study, we found that ADHD medications, particularly stimulants, generate more discussion on X in English than in Spanish, suggesting a higher interest or visibility of these treatments in English-speaking countries compared to Spanish-speaking ones. In both languages, stimulants such as methylphenidate are the most frequently mentioned medications, aligning with global prescription trends [20,47]. Moreover, a significant proportion of English-language tweets comes from patients, which could indicate greater active participation and awareness regarding ADHD diagnosis and treatment in these countries. This increased visibility of ADHD medications in English-language tweets corresponds with the higher prescription rates in English-speaking countries, such as the USA, compared to Spanish-speaking countries, like those in Latin America or Spain [13,47]. Several factors have been proposed to explain these differences, including disparities in access to diagnosis and treatment across regions [48,49,50] and political factors [51]. The immediate access to such information through social media analysis presents a major challenge and offers immense potential to be fully explored.

It is important to highlight that, in addition to their effectiveness in managing the core symptoms of ADHD, stimulant medications have been shown to impact patients’ quality of life and productivity positively [1,17,18,52]. In our study, the low frequency of mentions regarding side effects may reflect a favourable perception of the tolerability profile of these medications, as reported in the scientific literature [53,54]. However, this observation could also indicate a potential lack of awareness or knowledge about the adverse effects they may entail. For instance, Moran et al. concluded that stimulants could be associated with low (0.10% with methylphenidate; 0.21% with amphetamines) but significant adverse events, such as psychosis [55]; similar results were also described more recently by Hamard et al., also describing a higher risk of psychotic symptoms, with amphetamine rather than with methylphenidate [56]. This finding is particularly relevant, as our study found few explicit references to ADHD diagnosis in the tweets, while both languages, especially English, contained substantial content related to the misuse and trivialisation of these medications. This highlights the need for greater awareness of the potential risks associated with the misuse of these medications, particularly among adolescents and young adults, since there has been a growing number of news reports about drug misuse in this age group, especially during exam periods [57,58].

Based on this observation, the prevalence of tweets related to the inappropriate use of stimulant medications may not only indicate substance abuse but also point to unregulated access to these medications. Our analysis identified a substantial volume of requests and offers for these medications in both languages, supporting the hypothesis of an active illicit market. This issue is not isolated, as previous studies have demonstrated the utility of social media in monitoring the consumption of substances such as alcohol and the misuse of opioids [30,31]. Therefore, these findings highlight the importance of considering more robust pharmacovigilance strategies. In this regard, studies like that of Song et al. have proposed using social media as a potential tool for pharmacovigilance due to the immediacy and broad access to information it offers, compared to traditional methods [59].

The dissemination of accurate scientific information on social media could be a key strategy to mitigate the inappropriate use of these medications. While some articles discuss the effective dissemination of medical content on social media [60], others emphasise the need to optimise the quality, reach, and impact of such strategies [61]. Our study suggests that patients are the most frequent group posting tweets in English, highlighting the relevance of educating this population segment about the risks associated with misuse. In this regard, providing evidence-based information on X could be highly beneficial, as it would enable patients to make more informed decisions and increase their awareness of the dangers of using non-prescribed stimulants. For healthcare professionals, remaining attentive to the content circulating on social media may offer useful insights into patients’ concerns and perceptions. Bringing these discussions into the clinical setting could help counter misinformation, improve guidance, and contribute to a stronger physician–patient relationship through open dialogue and shared decision-making. The increasing presence of healthcare professionals on X, particularly in Spanish-language tweets, might therefore represent a positive step toward promoting a more informed and evidence-based online environment. Nonetheless, further studies are needed to corroborate these hypotheses, and our findings should be viewed as a modest step forward in this direction.

The findings of this study should be interpreted in light of several limitations. First, we included only tweets in English and Spanish, which may limit the representation of global linguistic diversity. In addition, there was a considerable imbalance in the proportion of tweets by language, with approximately 90% in English and 10% in Spanish [62]. While this asymmetry may introduce bias in cross-linguistic comparisons, it mirrors broader language trends on the X platform, where English is the most frequently used language globally. The proportion of Spanish tweets in our dataset is consistent with this global share [62]. More importantly, a central limitation of our study is that the comparisons were performed by language rather than by country. This approach may introduce bias, as English- and Spanish-speaking users belong to countries with different healthcare systems, policies, and prescription practices. Because the dataset only includes tweets up to 2022 and geolocation metadata was largely unavailable, only a very small number of users explicitly provided their location, which prevented us from conducting meaningful geographic stratification. Future research could address this limitation by combining social media data with geotagged information, user-provided metadata, or external databases of prescription and health system characteristics, in order to enable cross-country comparisons and reduce this potential bias.

The findings of this study should be interpreted in light of several limitations. First, we included only tweets in English and Spanish, which may limit the representation of global linguistic diversity. In addition, there was a considerable imbalance in the proportion of tweets by language, with approximately 90% in English and 10% in Spanish. While this asymmetry may introduce bias in cross-linguistic comparisons, it mirrors broader language trends on the X platform, where English is the most frequently used language globally, followed by Japanese and Spanish [62]. The proportion of Spanish tweets in our dataset is consistent with this global share. The overrepresentation of English-language tweets could also reflect a greater volume of public discourse surrounding ADHD in English-speaking countries, which may be linked to higher diagnosis rates and prescription levels, as discussed previously. Future research could address this limitation by applying stratified sampling strategies or including additional languages to support more balanced cross-cultural comparisons.

Moreover, because the dataset only includes tweets up to 2022, geolocation metadata was largely unavailable, and only a very small number of users explicitly provided their location, preventing us from performing meaningful geographic stratification. Also, some of the medications analysed are used to treat other conditions, like hypertension, highlighting the need for more precise identification of diagnoses mentioned in the tweets to improve the accuracy of the results. We also acknowledge that the exclusion of non-approved substances, such as modafinil or illicit stimulants, limits the comprehensiveness of the analysis, particularly from a pharmacovigilance perspective. Furthermore, our analysis focused primarily on pharmacological categories related to ADHD, and while some of these indirectly touch on issues like tolerance or drug combinations, we did not include broader clinical dimensions such as comorbid neurodevelopmental or psychiatric disorders, important aspects that warrant exploration in future research. In addition, although the machine learning models achieved a mean F1 score above 0.72 across all categories, indicating strong alignment with manual coding, we recognise that some degree of error, particularly in nuanced categories such as trivialisation or sarcasm, remains unavoidable. It is also important to note that engagement metrics, such as likes and retweets, may be influenced by automated accounts (bots); while these appear to be decreasing in health-related content [63], their potential presence should be considered when interpreting amplification. Additionally, it is possible that a significant portion of marginalised populations or older adults may not have access to X, potentially excluding these groups from the analysis.

Nonetheless, our study presents several strengths that contribute to the robustness of the findings. First, the analysis covers tweets spanning 16 years, providing a comprehensive view of trends and discussions over an extended period. Also, we classified the tweets by user type, allowing for a more nuanced understanding of who engages in these conversations, such as patients, healthcare professionals, and others. Third, by analysing tweets in both English and Spanish, we were able to highlight important linguistic differences in the discussion of ADHD medications, offering insights into varying perspectives worldwide. Additionally, this study offers valuable insights into the information circulating online about ADHD pharmacological treatments, information that many patients are exposed to and may carry with them but often do not bring up during clinical encounters. Factors such as social desirability bias can lead patients to withhold concerns or questions simply to align with perceived expectations from their clinician. By understanding the topics and perceptions prevalent in social media discourse, clinicians can proactively raise these issues in consultations, creating opportunities to address misconceptions, clarify doubts, and improve physician–patient communication. Moreover, identifying the presence of advocacy voices, healthcare professionals, and healthcare institutions underscores how social media can serve as both a powerful channel for disseminating evidence-based information and a platform vulnerable to misinformation. Recognising and addressing these dynamics creates opportunities for designing early detection strategies and evidence-based interventions directly within social media spaces.

## 5. Conclusions

Our study highlights the value of X as a tool for understanding public perceptions of ADHD medications. Beyond the predominance of stimulants mentioned, the presence of inappropriate use and trivialisation underscores the need for better public education on potential risks. Monitoring social media can offer clinicians deeper insights into patient concerns, fostering more effective communication and adherence, while also informing regulatory strategies and targeted health interventions on digital platforms.

## Figures and Tables

**Table 1:** This table summarises the main categories used in the tweet classification process, including concise definitions and representative examples. Categories were assigned only when tweets explicitly contained the relevant content, and ambiguous cases were excluded. Where possible, user profiles were cross-checked (e.g., to confirm healthcare professional status), but the primary classification criterion was always the tweet’s textual content.

Variable	Category and Explanation	Example
User type	**Patient**: Tweets where the author explicitly identified themselves as someone diagnosed with ADHD or currently using ADHD medications, sharing their personal experiences, struggles, or reflections related to treatment.	I was off work for 5 months. Going to counselling and seeing an OT for my cognitive challenges. Diagnosed with ADHD started vyvanse and was able to do a gradual return to work. My concentration was shot. My memory was now terrible. My once so capable brain was not familiar now.
User type	**Healthcare professionals:** Tweets written by individuals who explicitly self-identified as healthcare providers, usually discussing prescribing practices, clinical experiences, or medical observations about ADHD treatments. When possible, we also cross-checked the user’s biography for references to professional roles such as ‘Dr,’ ‘nurse,’ ‘therapist,’ or similar identifiers to support the classification.	I can’t imagine prescribing someone Dexedrine without an ADHD diagnosis and something like maybe narcolepsy. To put it in perspective Hunter S Thompson used to write frequently about Dexedrine 50+ years ago it’s an old drug with worse side effects than modern alternatives.
User type	**Healthcare institutions**: Tweets from verified or institutionally branded accounts, such as public health organisations, medical associations, or clinics, providing general medical advice, warnings, or news about ADHD medications.	Methylphenidate: safe and effective use to treat ADHD: Updated guidance to use methylphenidate to safely and…
Content	**Efficacy:** Tweets describing perceived effectiveness of ADHD medications, based on the author’s personal or observed experiences with symptom improvement or treatment response.	Concerta really works for me and it lasts for 8 h 😁 I would recommend talking to your therapist about trying it out!
Content	**Inappropriate use:** Tweets explicitly describing non-therapeutic or risky consumption patterns—for example, combining ADHD medications with alcohol or illicit drugs, or using them episodically to stay awake for long hours.	a lot of people have been asking me how I manage 2 tweet around the clock ⏰ amphetamine methylphenidate dextroamphetamine + red bull I popped Concerta a Vicodin Adderall drunk some whiskey Vodka smoked 3 blunts smoked a black…and now.
Content	**Side effects:** Tweets detailing negative physiological or psychological reactions to ADHD medications, including tolerability issues, physical symptoms, or emotional effects.	Concerta made me feel like a zombie. Vyvanse made my joint problems worse…
Content	**Psychiatric diagnosis:** Tweets that explicitly mentioned ADHD, either in relation to other psychiatric conditions, comorbidities, or the combined effects of ADHD and non-ADHD medications.	I was on Paxil now I’m on Sertraline for my anxiety. For my ADHD I was on Strattera & Intuniv those failed so now I’m on Vyvanse. I believe the Vyvanse and/or the Sertraline are causing me to have mood swings and anx. atks. though my anxiety and concentration is a little better
Content	**Economic and legal activities:** Tweets describing financial or insurance barriers to accessing medications, price concerns, or navigating regulatory steps such as titration or authorisation processes.	Oh God I switched from Concerta to Jornay PM and the insurance made me step titrate for the first time in 10 years and it was hellish. Like yeah lets take 30% of this drug I need daily. That will work
Content	**Advocacy:** Tweets advocating for specific treatments, sharing epidemiological warnings, or explaining pharmacological mechanisms to raise awareness or caution.	Observational non randomised data (use caution) show higher risk of #psychosis if #ADHD patients provided amphetamine-based medicines (#Adderall & #Vyvanse) instead of meds based on methylphenidate (#Ritalin or #Concerta)
Content	**Request and offer:** Tweets explicitly requesting or offering ADHD medications, either through formal channels or illicitly.	Buy strattera online… ADHD medications vyvanse. Click Here To Enter…
Content	**Trivialisation:** Tweets using ADHD medications in jokes, memes, or sarcastic remarks, often reflecting cultural trivialisation or stereotyping of ADHD treatment.	Hey Donald how’s that addiction to Adderall going?

**Table 2:** Number of tweets, ratio like/tweet and retweet/tweet classified by stimulant and non-stimulant drugs used in attention deficit hyperactivity disorder (ADHD).

	Total Original Tweets (English + Spanish)				English Original Tweets				Spanish Original Tweets			
Drug	n (Frequency)	% (Percentage)	Ratio Like:Tweet	Ratio Retweet:Tweet	n (Frequency)	% (Percentage)	Ratio Like:Tweet	Ratio Retweet:Tweet	n (Frequency)	% (Percentage)	Ratio Like:Tweet	Ratio Retweet:Tweet
**Stimulants**	183,571	74.78	97.68	16.33	166,726	75.21	106.98	17.81	16,845	70.86	5.65	1.74
Amphetamine	14,136	5.76	43.30	10.34	12,073	5.45	50.02	11.89	2063	8.68	3.99	1.30
Dextroamphetamine	43,779	17.83	374.25	61.59	43,457	19.60	376.97	62.03	322	1.35	6.77	2.40
Lisdexamfetamine	11,432	4.66	21.69	1.90	9437	4.26	26.08	2.24	1995	8.39	0.96	0.26
Methylphenidate	114,224	46.53	6.02	1.17	101,759	45.90	5.94	1.07	12,465	52.44	6.65	2.04
**Non-stimulants**	61,896	25.22	13.49	2.87	54,969	24.79	15.04	2.87	6927	29.14	1.18	2.87
Atomoxetine	41,007	16.71	1.85	0.35	37,782	17.04	1.91	0.25	3225	13.57	1.12	1.55
Guanfacine	8918	3.63	1.57	0.19	7310	3.30	1.84	0.19	1608	6.76	0.38	0.19
Viloxazine	1462	0.60	0.88	0.10	1438	0.65	0.88	0.09	24	0.10	0.96	0.83
Clonidine	10,509	4.28	70.75	15.33	8439	3.81	87.64	17.37	2070	8.71	1.91	7.03
Total	245,467	100			221,695	100			23,772	100		

## Data Availability

The raw data supporting the conclusions of this article will be made available by the authors on request.

## Supplementary Materials

The following supporting information can be downloaded at https://www.mdpi.com/article/10.3390/healthcare13192487/s1. Table S1: List of keywords used to obtain tweets that mentioned any of the pharmacological drugs approved for ADHD treatment; Figure S1: Visual summary of methodology and main results.
