# Supplementary material for: Unveiling Social Media Content Related to ADHD Treatment: Machine Learning Study Using X’s Posts over 15 Years
Source: Healthcare (Basel). 2025 Sep 30;13(19):2487. doi: 10.3390/healthcare13192487 (PMC12524690; doi:10.3390/healthcare13192487)

Supplementary Material

Table S1. List of keywords used to obtain tweets that mentioned any of the pharmacological drugs approved for ADHD treatment

NAME (Spanish)	NAME (English)	BRAND NAMES (Spanish)	BRAND NAME (English)
Metilfenidato	Methylphenidate	Rubifén, Medicebrán, Dextro-Metilfenidato, Medikinet, Equasym, Concerta, Ritalin	AptensioXR, Azstarys, Concerta, Contempla XR-ODT, Daytrana, Dexmethylphenidate hydrochloride, Focalin, Focalin XR, Jornay PM, Metadate CD, Methylin, Methylin ER, Quillichew ER, Quillivant XR, Relexxii, Ritalin
Lisdexanfetamina	Lisdexamfetamine	Elvanse	Vyvanse
Dextroanfetamina	Dextroamphetamine		Adderall, Dyanavel, Mydayis, ProCentra, Zenzedi, Dexedrine
Anfetamina	Amphetamine		Adzenys ER, Adzenys XR-ODT, Dyanavel XR, Evekeo, Evekeo ODT, Amphetamine Sulfate
Guanfacina	Guanfacine		Intuniv
Clonidina	Clonidine	Catapresan	Kapvay, Nexiclon XR
Atomoxetina	Atomoxetine	Strattera, Dezaprex, Atamax	Strattera
Viloxazina	Viloxazine		Qelbree

Figure S1: Visual summary of methodology and main results:

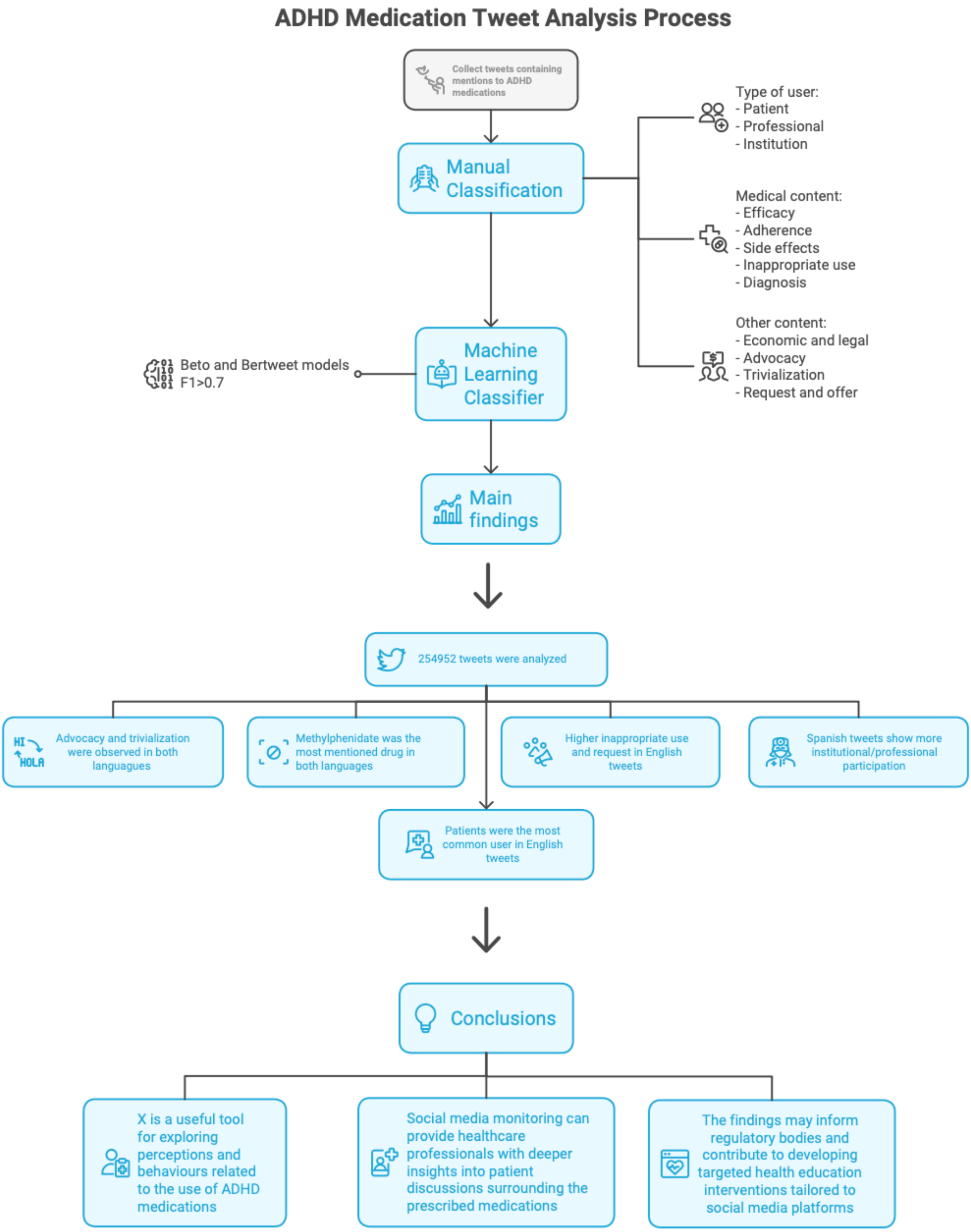

Supplement: Supplementary file 1 [file healthcare-13-02487-s001.zip › healthcare-3821890-supplementary.pdf]
